# Laminin Peptide-Immobilized Hydrogels Modulate Valve Endothelial Cell Hemostatic Regulation

**DOI:** 10.1371/journal.pone.0130749

**Published:** 2015-06-19

**Authors:** Liezl Rae Balaoing, Allison Davis Post, Adam Yuh Lin, Hubert Tseng, Joel L. Moake, K. Jane Grande-Allen

**Affiliations:** Department of Bioengineering, Rice University, Houston, TX, 77005, United States of America; University of California, San Diego, UNITED STATES

## Abstract

Valve endothelial cells (VEC) have unique phenotypic responses relative to other types of vascular endothelial cells and have highly sensitive hemostatic functions affected by changes in valve tissues. Furthermore, effects of environmental factors on VEC hemostatic function has not been characterized. This work used a poly(ethylene glycol) diacrylate (PEGDA) hydrogel platform to evaluate the effects of substrate stiffness and cell adhesive ligands on VEC phenotype and expression of hemostatic genes. Hydrogels of molecular weights (MWs) 3.4, 8, and 20 kDa were polymerized into platforms of different rigidities and thiol-modified cell adhesive peptides were covalently bound to acrylate groups on the hydrogel surfaces. The peptide RKRLQVQLSIRT (RKR) is a syndecan-1 binding ligand derived from laminin, a trimeric protein and a basement membrane matrix component. Conversely, RGDS is an integrin binding peptide found in many extracellular matrix (ECM) proteins including fibronectin, fibrinogen, and von Willebrand factor (VWF). VECs adhered to and formed a stable monolayer on all RKR-coated hydrogel-MW combinations. RGDS-coated platforms supported VEC adhesion and growth on RGDS-3.4 kDa and RGDS-8 kDa hydrogels. VECs cultured on the softer RKR-8 kDa and RKR-20 kDa hydrogel platforms had significantly higher gene expression for all anti-thrombotic (ADAMTS-13, tissue factor pathway inhibitor, and tissue plasminogen activator) and thrombotic (VWF, tissue factor, and P-selectin) proteins than VECs cultured on RGDS-coated hydrogels and tissue culture polystyrene controls. Stimulated VECs promoted greater platelet adhesion than non-stimulated VECs on their respective culture condition; yet stimulated VECs on RGDS-3.4 kDa gels were not as responsive to stimulation relative to the RKR-gel groups. Thus, the syndecan binding, laminin-derived peptide promoted stable VEC adhesion on the softer hydrogels and maintained VEC phenotype and natural hemostatic function. In conclusion, utilization of non-integrin adhesive peptide sequences derived from basement membrane ECM may recapitulate balanced VEC function and may benefit endothelialization of valve implants.

## Introduction

The endothelium plays an essential role in maintaining homeostasis in cardiovascular tissue. These cell monolayers act as a barrier between the circulating blood and the underlying tissue, produce and release various growth and vasomotor factors, and initiate inflammatory and clotting mechanisms in response to injury and disease [1,2]. Although the function of vascular endothelial cells has been thoroughly characterized, recent work has shown that cardiac valve endothelial cells (VECs) have distinct phenotypes and distinct gene and protein expression compared to vascular endothelial cells [3–5]. Additionally, VECs produce anti-thrombotic and thrombotic proteins differently from vascular endothelial cells, and can be affected by environmental changes in aortic valve tissue with respect to aging [5]. However, VECs still maintain characteristic endothelial functions, including signaling to the underlying valve interstitial cells (VICs) with vasoactive factors, and reducing extracellular matrix (ECM) degradation in the valve [6,7].

Given the interplay between VECs, VICs, and ECM, VECs and their regulation of valve homeostasis are highly sensitive to surrounding stimuli. For example, the presence of TGF-β and Notch-1 has been shown to promote VIC calcification, as well as to induce endothelial to mesenchymal transdifferentiation and influence VEC plasticity [8–10]. VEC dysfunction through pathogenic angiogenesis and imbalanced inflammatory and thrombotic protein regulation contribute to the progression of calcific aortic valve disease (CAVD) [5,11–13]. Myxomatous diseased valves have also been linked to thrombosis and endocarditis from disrupted valve endothelium [14,15]. Thus, the preservation of a healthy, functional valve endothelium is necessary for valve homeostasis and prevention of valve disease.

Various strategies have been investigated to promote the endothelialization of tissue-engineered heart valve scaffolds and implants to reduce thrombosis-related failures *in vivo*. However, heart valves are particularly challenging to endothelialize due to the high hemodynamic and mechanical forces experienced by valve tissues. Most approaches have had mixed success using vascular endothelial cells in combination with mechanical stimulation to promote endothelialization of decellularized valve tissues or mechanical valves [16–19]. Other studies have attempted to drive endothelial progenitor cells and mesenchymal stem cells to differentiate into endothelial cell lineage for endothelialization purposes [20–23]. However, little work has been done to evaluate differences or similarities in how these various cell types regulate hemostasis and perform their anti-clotting roles relative to native VECs [23,24].

This work investigates the thrombotic and anti-thrombotic behavior of primary porcine aortic VECs when cultured in the presence of various environmental stimuli. We have developed a platform utilizing poly(ethylene glycol) diacrylate (PEGDA) hydrogels and specific cell adhesive peptides to modulate the substrate stiffness and ECM presented on the surface to which the VECs are seeded. PEGDA-based hydrogels are bioinert polymers that can have tunable mechanical properties based on the molecular weight of the PEGDA used [25–27]. The acrylates on the surface of the gel are free to interact with desired adhesive peptides containing a thiol group [28,29]. Furthermore, VECs have been shown to have protein-dependent adhesion in previous studies [30]. To investigate the effects of ECM on VEC phenotype and hemostatic function, peptide sequences derived from laminin and fibronectin proteins were used. Laminins belongs to a family of heterotrimeric glycoproteins composed of combinations of α, β, and γ chains. Laminins in the basement membrane play an integral role in the formation of the basement membrane network [31]. The peptide motif RKRLQVQLSIRT (RKR) is found in the laminin-α1 heparin binding G-domain, and has previously been shown to promote strong cellular attachment activity [32,33]. The cell adhesive peptide RGDS is found in fibronectin and other ECM proteins, and is one of the most commonly used peptide sequences to promote cellular adhesion [28,34,35]. These studies will provide information about VEC hemostatic capacity and conditions necessary to maintain essential VEC functions *in vitro*, and potential strategies for endothelialization of cardiovascular implants.

## Materials and Methods

### Ethical approval

As described below for the platelet adhesion assay, platelet rich plasma (PRP) was isolated from whole blood drawn from healthy, adult volunteers. Written consent was obtained from each volunteer at the time of donation. The Institutional Review Board at Rice University approved the consent procedure and this specific protocol.

### PEGDA synthesis and hydrogel characterization

PEGDA hydrogels are non-immunogenic, mechanically tunable, and naturally prevent protein adsorption and cell adhesion unless they are chemically modified [25,27,34]. To control scaffold substrate rigidity, PEGDA hydrogels of molecular weights (MWs) 3.4, 8, or 20 kDa were used.

PEGDA was prepared following previously described methods [26,27]. Briefly, PEG powder with molecular weight of either 3.4, 8, or 20 kDa was acrylated by mixing 0.4 mmol of PEG (Sigma-Aldrich) with 0.016 mol of acrylolyl chloride and 0.8 mmol of triethylamine in anhydrous dichloromethane (DCM) under argon gas overnight. The PEGDA solution was washed and mixed with 2M K_2_CO_3_ and phase separated into aqueous phase, and dried with anhydrous MgSO_4_ to remove any residual solution. Next, the MgSO_4_ was filtered from solution, and PEGDA precipitated from the DCM and filtered with cold diethyl ether. The resulting PEGDA powder was tested with ^1^H-NMR to verify acrylation of PEG chains. PEGDA samples were stored at -20°C until use.

PEGDA hydrogels were polymerized by dissolving PEGDA powder of a particular MW in deionized H_2_O at 10% (w/v) with 45 mM of the photoinitiator, Irgacure 2959 (Ciba, Basel, Switzerland), and exposed to UV light for 5 min. on each side (365 nm, 10 mW/cm^2^). Once polymerized, the hydrogels were soaked in phosphate buffered saline (PBS, pH 7.4) at room temperature overnight to swell and remove excess photoinitiator. Hydrogels used for experiments were approximately 0.5–1.5 mm thick after swelling. Disks 22 mm in diameter were punched from the bulk hydrogels, placed into 12-well tissue culture plates, and seeded with VECs for experiments.

Compressive mechanical testing was performed on 22 mm diameter, 5 mm thick hydrogel disks made of each PEGDA MW using a Bose ElectroForce ELF 3200 (Eden Prairie, MN) system using a 1000 gram load cell (Bose). Each sample was compressed to 30% strain, and the resulting load was measured. The stress and strain was calculated at each time point, and plotted against each another. The compressive elastic modulus of each PEGDA sample was calculated as the slope of the linearly elastic stress-strain curve using Microsoft Excel (n = 5).

### ECM Adhesive Peptide Motifs

Both RKR and RGDS peptides were modified to include a cysteine at the N-terminus of each sequence, resulting in peptide sequences CRKRLQVQLSIRT and CRGDS. The cysteine introduced a thiol group at the end of each peptide chain that could react with the acrylate groups on the surface of the PEGDA hydrogels. The custom modified RKR and RGDS peptides were synthesized by American Peptide Company (Vista, CA), reconstituted in sterile dimethyl sulfoxide, and stored at -80°C until use.

To immobilize the peptide motifs onto the PEGDA hydrogels, 3 mM peptide solutions were added to the hydrogel surface and exposed to 5 min. of UV light and 45mM I2959 to initiate a thiol-ene reaction, covalently binding the desired peptide to the gel via click reaction [28,36]. The hydrogels were vigorously washed with 0.05% Tween-20 detergent and PBS to remove unbound peptides and solvent solution. Thiol-PEG-fluorescein (FITC, NanoCS, New York, NY) served as a negative adhesive substrate control capable of undergoing the thiol-ene reaction with hydrogels. Peptide immobilization and surface saturation was verified by measuring fluorescent signal (418 nm) from increasing concentrations (0-15mM) of thiol-PEG-FITC immobilized onto 3.4 and 20 kDa hydrogels using a spectrophotometer (SpectraMax M2, Molecular Devices, Sunnyvale, CA) (n = 3 per concentration) (S1 Fig).

### Valvular Endothelial Cells

VECs were harvested, on several occasions, from fresh aortic heart valves dissected from 6-month old porcine hearts purchased from a local commercial abattoir (Fisher Ham and Meats, Spring, TX). Each batch of isolated VECs was pooled from three aortic valves (harvested from three porcine hearts) to promote biological variation. VEC isolation and purification procedures followed a combination of previously described methods [4,5,37]. Briefly, each dissected aortic leaflet was rinsed in sterile 5% Penicillin Streptomycin/PBS, and soaked in an enzyme digestion composed of 2 U/mL of dispase (Stem Cell, Vancouver, Canada), and 60 U/mL of collagenase type II (Worthington Biochemical Corporation, Lakewood, NJ) at 37°C for 1 hr. After the enzyme digestion, the VECs were gently scraped off each leaflet surface by rolling a swab tip across both sides of the leaflets. The cells were rinsed off the swab with media, centrifuged, and then resuspended in media and plated. All VEC cultures used EGM-2 media supplemented with growth factor bullet kit (hydrocortisone, FBS, VEGF, hEGF, hFGF-B, R^3^-IGF-1, ascorbic acid) and 1% v/v Penicillin Streptomycin solution (PenStrep, Lonza, Walkerville, MD). After the initial passage, VECs were purified from any contaminant interstitial cells via magnetic bead cell isolation (CELLection Pan Mouse IgG Kit, Invitrogen, Carlsbad, CA) using mouse anti-porcine CD31 antibody (AbD Serotec, Oxford, UK). VECs were cultured in a humidified incubator (37°, 5% CO_2_) until they reached 90% confluence, with media changed every 2–3 days. VECs from passages P2-P4 were used in the following experiments.

### Experimental Groups

To assess the effects of substrate stiffness and ECM on VEC phenotype and behavior, VEC were seeded at a cell density of 250 cells/mm^2^ onto combinations of the 10% w/v PEGDA hydrogels composed of three different PEGDA MWs (3.4, 8, and 20 kDa) and immobilized with either modified ECM adhesive peptides (RKR or RGDS). Thus, six microenvironment culture conditions were examined. Uncoated PEGDA hydrogels of each PEGDA MW were used as negative controls, whereas tissue culture treated polystyrene (TCPS) dishes were used as baseline controls for VEC attachment. Cells were cultured for 5–8 days on the peptide-immobilized gels until 90% confluence was reached. Evaluation of cells in confluent culture was performed as described below. VEC adhesion and proliferation was monitored daily with light microscopy.

### Immunofluorescence and Quantitative RT-PCR

Immunofluorescence was performed on cells fixed in 4% paraformaldehyde. Mouse anti-CD31 (PECAM-1) (LCI-4, AbD Serotec), and mouse anti-α-smooth muscle actin (αSMA) (1A4, Abcam, Cambridge, MA) antibodies were used to verify that the VEC cultures demonstrated the endothelial phenotype with no interstitial cell contamination. Rabbit anti-laminin (Abcam) and mouse anti-fibronectin (A17, Abcam) were used to observe ECM production by VECs. Rabbit anti-porcine von Willebrand factor (VWF, Abcam) was used to visualize VWF produced by VECs. Secondary antibodies conjugated with Alexa Fluor (Invitrogen) were used after primary antibody incubation. Fluorescent imaging was performed using a Zeiss LSM 5Live Confocal Microscope (Zeiss, Oberkochen, Germany).

Once confluent, VECs were trypsinized and scraped from their substrates, centrifuged, and lysed with Trizol Reagent (Invitrogen). Using a series of ethanol washes and centrifugations, mRNA from the VEC samples were collected. The mRNA was reverse transcribed into cDNA using PrimeScript 1^st^ strand cDNA Synthesis Kit (Takara Bio, Otsu, Japan). The cDNA samples were stored at -80°C until use. Using the 2X QuantiTect SYBR Green PCR Master Mix (Clontech, Mountain View, CA), quantitative RT-PCR (qRT-PCR) on the cDNA was performed to assess differences in gene expression levels for the above mentioned anti-thrombotic and thrombotic proteins between VECs cultured in the various platforms. Sample size was 3–4 experimental cultures per platform, with each sample then measured in triplicate technical replicates for PCR. GAPDH gene was used as the housekeeping gene, and sample group gene expression levels were normalized to the corresponding expression levels of the TCPS seeded VECs (see S1 Table for primer sequences).

### VEC Histamine Stimulation

Previous work has shown that the addition of histamine to human umbilical vein EC cultures *in vitro* effectively initiates EC secretion and anchorage of hyper-thrombotic ultra-large VWF (ULVWF) multimer chains previously stored in Weibel-Palade bodies at the cell membrane, while not affecting EC expression and release levels of the VWF cleaving enzyme ADAMTS-13 [38,39]. The measurable quantities of total VWF protein or inactivated, cleaved VWF fragments (VWF 140-kDa or VWF 176-kDa) in the solution are commonly used to quantify the functionality and capacity of ADAMTS-13 enzymatic cleavage of VWF. VECs were incubated with serum-free EGM-2 media (with 1% v/v of insulin-transferring selenium A (Sigma-Aldrich) and 1% BSA w/v) containing 100 μM of histamine at 37°C for 2 minutes, and then rinsed with PBS. After, cells were incubated in complete EGM-2 media for 10 min. Then, media supernatants were collected into 10 mM of EDTA and analyzed by sandwich ELISA for either total VWF antigen or VWF 140-kDA fragments.

### Measurement of total VWF and VWF 140-kDa fragments

A sandwich ELISA was performed to measure cleaved and uncleaved VWF from histamine stimulated VECs (n = 3, with each sample then measured in triplicate technical replicates) following previously described methods [5,39]. Maxisorb 96-well plates (Nunc, Penfield, NY) were coated with 1 μg/mL of polyclonal rabbit anti-porcine VWF antibody (Abcam) in a Coating Solution buffer (KPL, Gathersburg, MD) overnight at 4°C. The wells were then blocked with 1% w/v BSA/PBS solution for 1 hr at 37°C. Samples of cell media supernatant or porcine plasma were diluted with 1% BSA/PBS solution and incubated in the wells for 1 hr at 37°C. Next, the wells were washed 3x with 1x Washing Solution (KPL), and incubated with 1 μg/mL of detection antibody for mouse anti-porcine full length VWF protein monoclonal antibody (2Q2134, Abcam) or mouse anti-human VWF 140-kDa fragment antibody (amino acids L^1591^-Y^1605^) for 1 hr at 37°C [39,40]. Afterwards, the wells were again washed 3X with Washing Solution, and incubated with 1 μg/mL of peroxidase-labeled anti-mouse IgG (KPL) for 1hr at RT. The wells were washed 4x with Washing Solution and then incubated with SureBlue Reserve TMB peroxidase solution to expose the peroxidases on the bound VWF-detection antibodies. The reaction was stopped with the addition of TMB Stop Solution (KPL), and the 450 nm absorbance of each well was read using a spectrophotometer. Serial dilutions of porcine plasma (Animal Technologies, Tyler, TX) was used to create a standard curve, with the assumption of 10 μg/mL of VWF present per mL of pooled plasma [41].

### Platelet Adhesion Assay

To assess how the microenvironmental conditions affect VEC capacity to activate and adhere live platelets, dyed platelets were added to VEC-seeded constructs and quantified following previously described methods by Xu et al [42]. Platelet rich plasma (PRP) was isolated from whole blood drawn from healthy, adult volunteers following a protocol approved by the Institutional Review Board at Rice University. The PRP was treated with prostacyclin (0.5 μg/mL of PRP) to prevent platelet activation during subsequent dyeing and washing steps. The platelets were isolated from the PRP, resuspended in Tyrode’s buffer, and dyed with Sudan B Black solution (5% w/v) for 1 hour. The dyed platelets were washed 3x with PBS.

The VECs seeded on hydrogel constructs and TCPS (n = 3 experimental replicates per platform) were gently washed with PBS to remove residual media. Next, 4x10^6^ platelets were added to each sample and allowed to incubated at 37°C for 30 minutes. The constructs were then moved to a new well plate to remove any unadhered platelets on the bottom of the gel and then gently washed 3x with PBS. Next, 400 μL of DMSO was added to each well to lyse the bound platelets and release the SBB dye. The absorbance of the supernatant for each sample was read in duplicate at 595 nm using a spectrophotometer. The total number of adhered platelets was calculated based on a standard curve made from serial dilutions of the prepared dyed platelet stock solution. This platelet adhesion assay was subsequently performed on VECs stimulated with histamine (as described above) to assess the thrombogenicity of activated VECs and their released VWF.

### Statistics

All experiments were performed in duplicate with the sample sizes and number of technical replicates noted above. Combined data from both duplicates are presented as means with standard errors of means (SEM). ANOVA statistics and Tukey *post hoc* tests were performed using JMP statistical software (SAS, Cary, NC) to compare the differences between the peptide-gels used as platforms for VEC cultures. P-values < 0.05 were considered statistically significant.

## Results

### PEGDA hydrogels have tunable mechanical properties

The compressive moduli for the 3.4, 8, and 20 kDa 10% w/v hydrogels were 132.0 ± 5.9 kPa, 34.6 ± 3.2 kPa, and 7.3 ± 1.1 kPa, respectively. Increasing the MW of PEGDA hydrogels resulted in a decrease of the bulk stiffness of the gels.

### ECM peptides influence VEC adhesion and proliferation on PEGDA hydrogels of different rigidities

**Fig 1:**
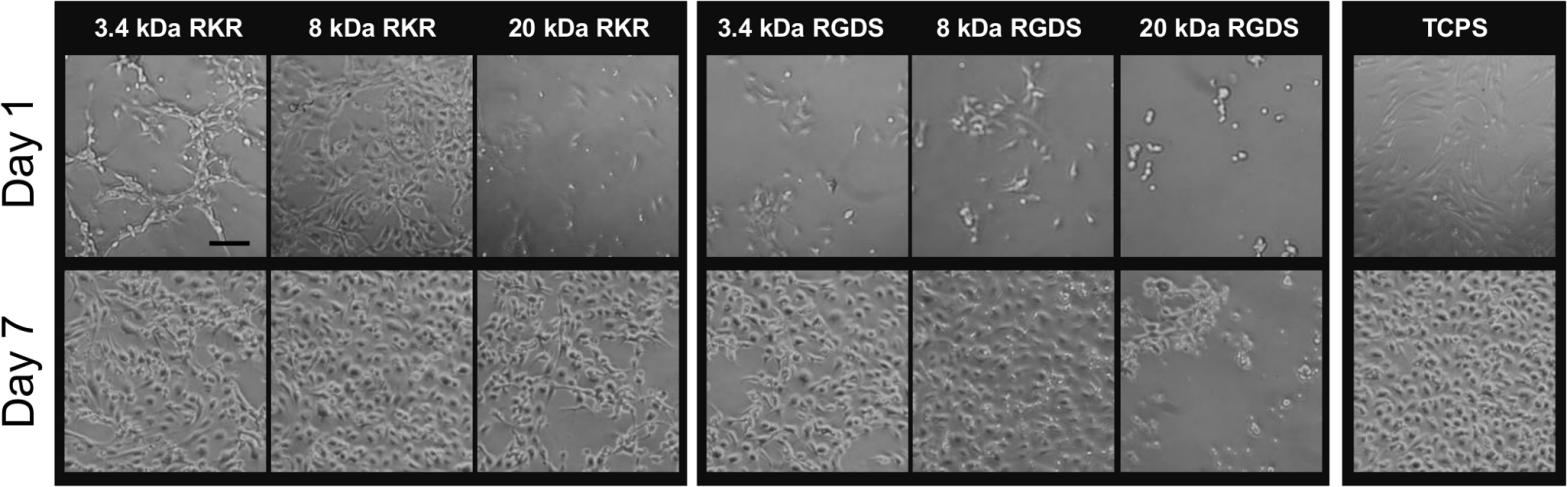
VECs adhere onto hydrogel scaffolds immobilized with extracellular matrix-derived peptides. Representative images of valve endothelial cell (VEC) adhesion and growth when seeded on combinations of 3.4, 8, or 20 kDa molecular weight PEGDA hydrogels with immobilized CRKRLQVQLSIRT (RKR) (first panel) or CRGDS (RGDS) (second panel) and on tissue culture treated polystyrene (TCPS) on days 1 and 7. Scale bar = 100 μm.

VECs seeded onto the laminin-derived RKR-coated constructs were found to adhere onto all hydrogel rigidities by 24 hrs. As shown in Fig 1, after 24 hours, VECs attained 50% and 68% confluence on 3.4 and 8kDa hydrogels, respectively. The cells proliferated on all the RKR samples, reaching 100% confluence within 3 days on the RKR-8 kDa PEGDA hydrogels. Within 7 days of culture, VEC monolayers were 100% confluent on the RKR-3.4 kDa and RKR-8 kDa hydrogels, whereas VECs on the RKR-20 kDa gels were at approximately 90% confluence (Fig 1).

Although VECs adhered to the RGDS-coated hydrogels within 24 hrs, the cells were observed to spread and proliferate slower compared to VECs on the TCPS control and RKR based hydrogel samples, with only 19% and 12% confluence respectively on the 3.4 and 8kDa gels shown in Fig 1. After 7 days of culture, a 100% confluent monolayer was present on the RGDS-3.4 kDa gels, whereas the VECs on the RGDS-8 kDa gels were only at ~85–90% confluence. The VECs on the RGDS-20 kDa hydrogels, however, did not proliferate and were only weakly adherent on the constructs. VEC samples seeded on the soft RGDS-20 kDa hydrogels were therefore excluded from the following experiments.

### Peptide-hydrogel platforms support VEC monolayer formation and ECM production

**Fig 2:**
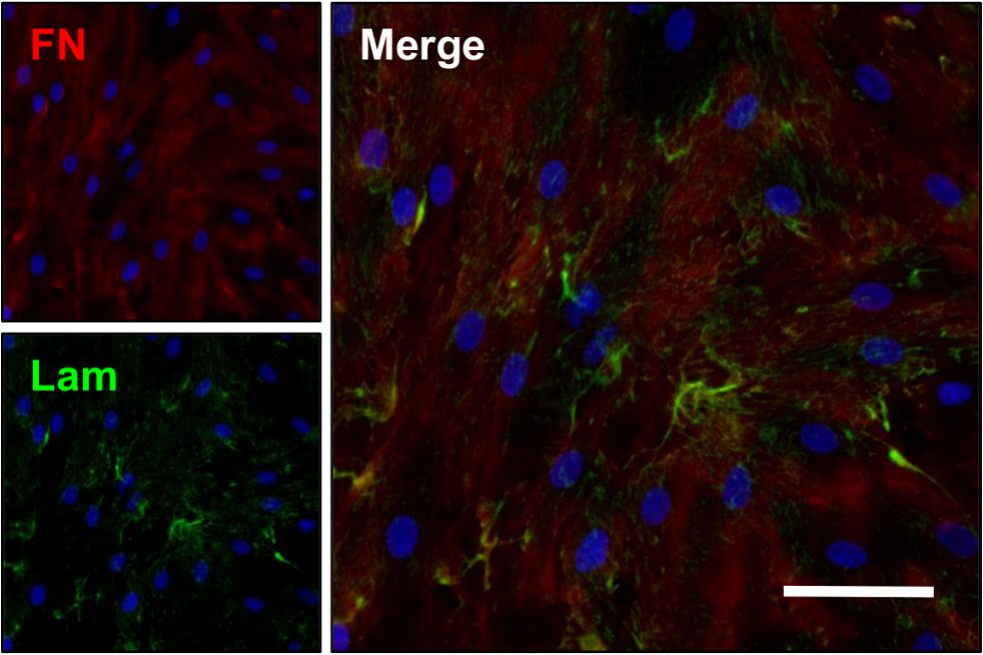
VECs on peptide-coated hydrogels produce extracellular matrix proteins. Extracellular matrix proteins fibronectin (FN, red) and laminin (Lam, green) present throughout the cell layer of VECs cultured on RKR-8 kDa scaffolds after 7 days. Scale bar = 50 μm.

VEC cultures on all substrate combinations maintained their characteristic cobblestone morphology (Fig 1), staining positively for CD31 (PECAM-1) and VWF, and were negative for VIC marker αSMA. After 7 days of culture, VEC samples produced their own ECM, specifically laminin and fibronectin proteins (Fig 2).

### VEC gene expression of hemostatic proteins affected by microenvironment

**Fig 3:**
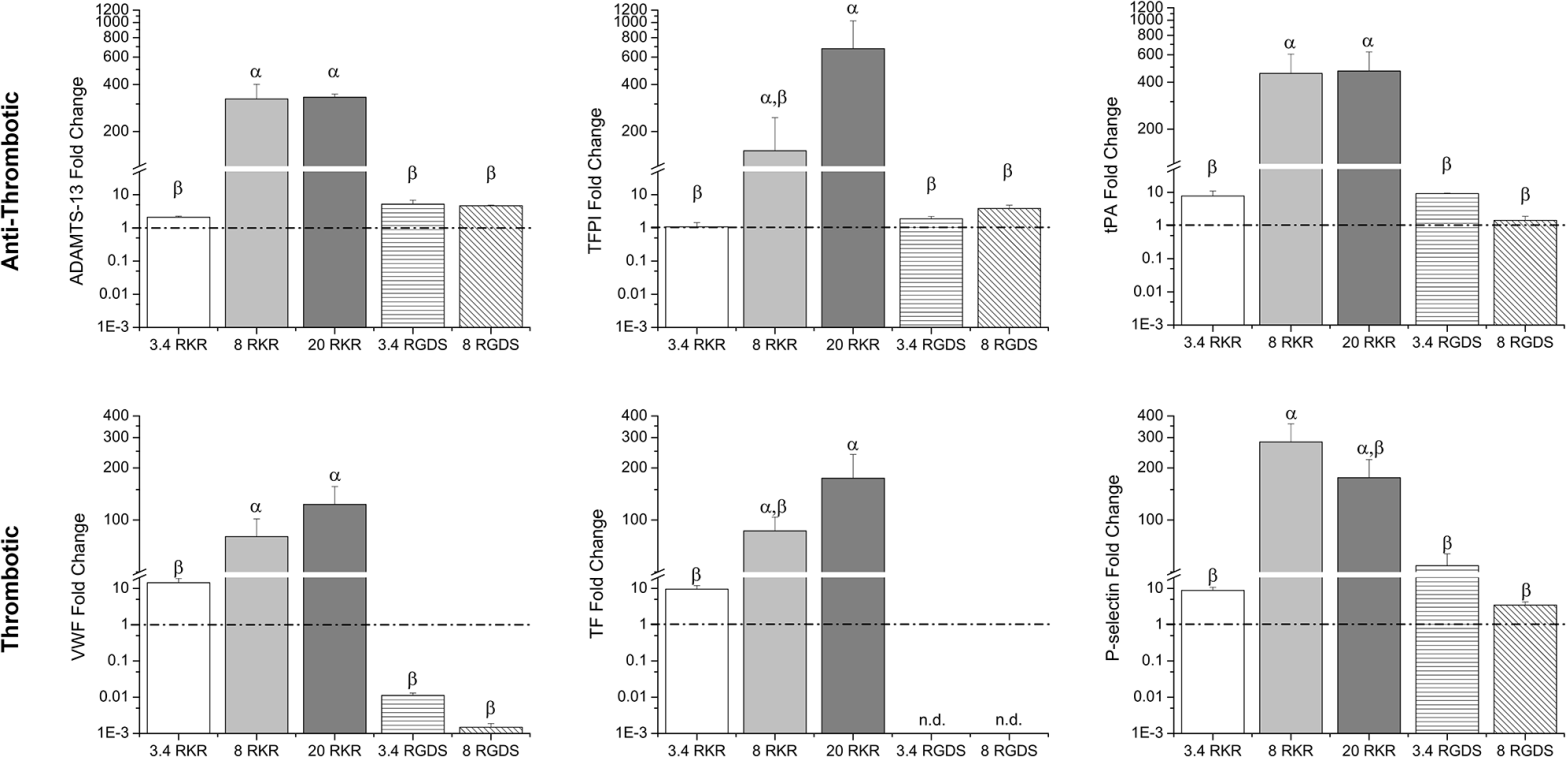
VEC hemostatic-related protein gene expression. VEC gene expression of anti-thrombotic related proteins [A disintegrin and metalloproteinase with a thrombopondin type I motif, member 13 (ADAMTS-13), tissue factor pathway inhibitor (TFPI), and tissue plasminogen activation (tPA)] and thrombotic related proteins [von Willebrand factor (VWF), tissue factor (TF), and P-selectin)] when cultured on combinations of different molecular weight formulations (3.4, 8, and 20 kDa) of hydrogels and adhesive ligands (RKR or RGDS) relative to VECs cultures on TCPS (indicated by dashed line). Groups not connected by same symbols are significantly different. p < 0.05.

Quantitative RT-PCR (qRT-PCR) analysis was used to assess the VEC gene expression of various anti-thrombotic and thrombotic proteins. With the gene expression levels for each protein normalized relative to the TCPS VEC control, VECs cultured on RKR-8 kDa and RKR-20 kDa hydrogels had increased gene expression for all tested proteins relative to other groups (Fig 3). In assessing gene expression for anti-thrombotic proteins, there were no differences in gene expression for ADAMTS-13 between VECs seeded on the RKR-8 kDa and RKR-20 kDa hydrogels; however, these cultures expressed significantly more ADAMTS-13 than VECs on RKR-3.4 kDa hydrogels, RGDS-3.4 kDa and RGDS-8 kDa hydrogels, and TCPS (80x v. the other groups, p<0.05). Similarly, the tPA expression of VECs on RKR-8 kDa and RKR-20 kDa constructs were greater than for VECs on all other groups (~75x v. the other groups, p<0.05). Furthermore, the gene levels for TFPI of VECs on RKR-20 kDa gels were greater than from those on RGDS-3.4 kDa and RGDS-8 kDa gels, RKR-3.4 kDa gels, and TCPS (p<0.05).

**Fig 4:**
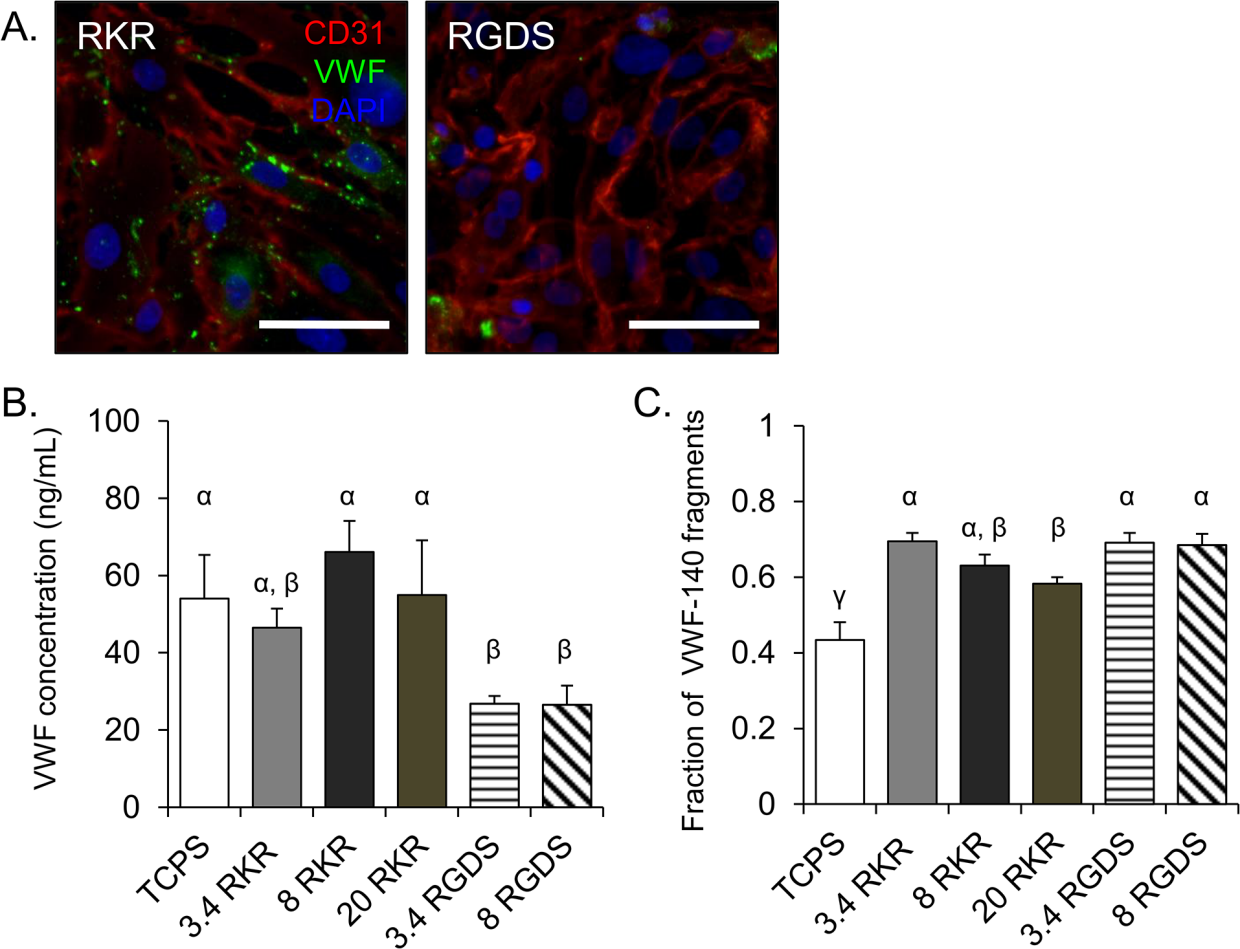
VECs produce, release, and actively cleave von Willebrand factor. (A) Non-stimulated VECs seeded on hydrogels (3.4 kDa) functionalized with RKR (left) or RGDS (right) stained for CD31 (red) and VWF (green). Nucleus was stained with DAPI (blue). Scale bar = 50 μm. (B) Quantification of rapidly released VWF and (C) the fraction of cleaved VWF-140 fragments from histamine-stimulated VECs cultured on different molecular weight hydrogels functionalized with RKR or RGDS, or TCPS. Groups not connected by same symbols are significantly different. p < 0.05.

Microenvironmental effects on VEC gene expression for thrombotic proteins were also assessed. The VECs seeded onto RKR-8 kDa and RKR-20 kDa hydrogels had significantly higher expression of VWF than the remaining groups (10x v. RKR-3.4 kDa; 1000x v. all RGDS groups, p<0.05). Immunofluorescent stains confirmed lower levels of VWF in VECs on RGDS-3.4 kDa gels than in VECs on RKR-3.4 kDa samples as well (Fig 4A). TF gene expression for the VEC group on RKR-20 kDa gels was greater than the RKR-3.4 kDa VEC group (15x, p<0.05); furthermore, neither of the VECs on RGDS-3.4 kDa and RGDS-8 kDa groups had any detectable levels of TF expression, and were excluded from statistical analysis for that gene. Gene expression for P-selectin was significantly higher in VECs on RKR-8 kDa gels than in VECs on the RGDS-3.4 kDa, RGDS-8 kDa samples, RKR-3.4 kDa gels, and TCPS (12x v. the other groups, p<0.05).

### Adhesive ligands influence VWF protein release, but do not affect ADAMTS-13 activity

VWF release and cleavage assays were performed on VECs cultured on each microenvironment combination and TCPS. Consistent with the qRT-PCR expression data, VECs cultured on RKR-8 kDa and RKR-20 kDa gels had the highest levels of VWF protein release among the tested microenvironments, though the RKR-coated groups overall showed no statistically significant difference in VWF protein release compared to TCPS (Fig 4B). The VECs on RGDS-3.4 kDa and RGDS-8 kDa groups had significantly lower VWF release than did VECs on the RKR-8 kDa and RKR-20 kDa gels and TCPS control (p<0.05).

To evaluate the VWF cleavage activity of ADAMT-13, ELISAs were performed to quantify the VWF cleavage byproduct, VWF-140 fragments (Fig 4C). There was no difference in the fraction of cleaved VWF-140 fragments between VECs cultured RKR-3.4 kDa, RKR-8 kDa, RGDS-3.4 kDa, and RGDS-8 kDa gels. However, the fraction of VWF-140 measured for the VECs on RKR-20 kDa samples was significantly lower than for the VECs on RKR-3.4 kDa and both RGDS-coated groups. The fraction of VWF-140 fragments by VECs on TCPS was significantly less than in all hydrogel based groups.

### VECs on hydrogels maintain capacity to adhere platelets when stimulated

**Fig 5:**
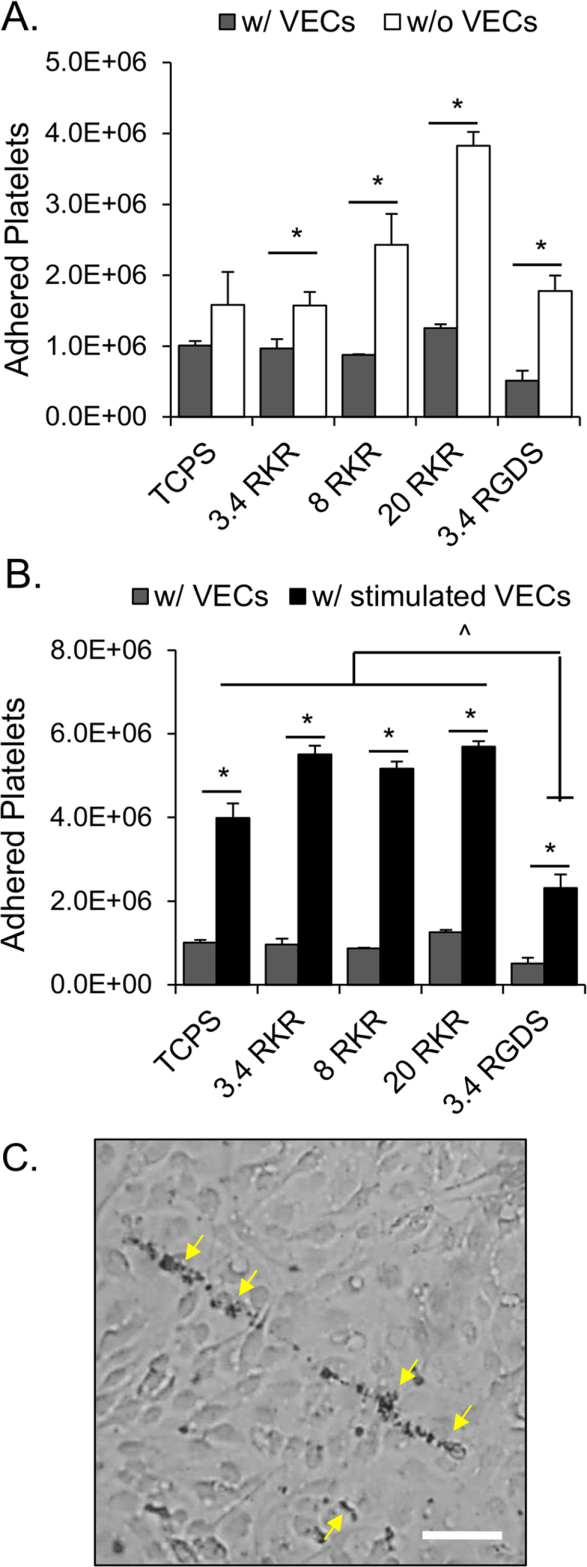
Histamine stimulated VECs on hydrogel based platforms are capable of promoting platelet adhesion. (A) Endothelialized peptide-immobilized hydrogel platforms significantly reduce platelet adhesion. Platelet adhesion on hydrogel scaffolds increases with increasing molecular weight PEGDA hydrogel. Platelet adhesion is significantly reduced on VEC-seeded peptide-coated hydrogels (gray bars) when compared to acellular peptide-coated constructs (white bars). * p <0.05. (B) Total adhered platelets onto stable and stimulated VECs cultured on TCPS and hydrogels with immobilized RKR or RGDS. Histamine stimulated VECs (black bars) of all culture condition promoted significantly more platelet adhesion when compared to the non-simulated VECs cultured on the same condition (gray bars). * p < 0.05. Platelet adhesions were significantly lower for the stimulated VECs on RGDS gels than for all other stimulated conditions. # p < 0.05. (B) Dyed platelets adhered onto anchored ultra-large VWF strings (yellow arrows) on the surface of histamine stimulated VECs cultured on a RKR-3.4 kDa hydrogel. Scale bar = 50 μm.

The total number of adhered platelets on each VEC-seeded hydrogel construct ranged between 0.5-1x10^6^ platelets, with no significant difference in platelet adhesion levels between the various peptide-coated hydrogel conditions. Platelet adhesion levels for the VEC-seeded hydrogels were significantly less than on their respective unseeded peptide-hydrogel combinations (Fig 5A) (p<0.05). The ratio of adhered platelets on cell-free RKR-coated hydrogels to VEC-seeded RKR-coated hydrogels increased with increasing MW of the hydrogel used (1.6x for 3.4 kDa, 2.8x for 8 kDa, and 3.0x for 20 kDa). As VECs seeded on RGDS-8 kDa had a tendency to lift off the hydrogel after histamine stimulation, and could not maintain adhesion throughout the platelet adhesion assay, the RGDS-8 kDa group was excluded from this experiment.

When VECs cultured on the hydrogel-peptide platform combinations and TCPS were stimulated with histamine, a significantly greater amount of platelets adhered to the stimulated VECs compared to their respective non-stimulated states (Fig 5B) (p<0.05). Dyed platelets were observed to adhere onto anchored ULVWF strings of stimulated VECs under light microscopy (Fig 5C). However, there were significantly less adhered platelets on the stimulated VECs on RGDS-3.4 kDa hydrogels than on all other conditions (0.5x v. RKR and TCPS groups, p<0.05).

## Discussion

VECs play important roles in maintaining valve tissue health. Due to their unique endothelial sub-phenotype, VECs have slightly different regulation of homeostatic functions than do vascular endothelial cells [3]. Among the many essential functions, the balance in VEC production of anti-thrombotic and thrombotic proteins is lost with age associated remodeling in the native valve environment [5]. Under pathological conditions, VEC expression for anti-thrombotic proteins is altered, and the ability to respond to injury is decreased [5,14,15]. Although several groups have studied vascular endothelial cells on biomaterials, the influence of biomaterials on VECs is still unknown [43]. Previous studies show PEG-coated implants have desirable thromboresistant characteristics, however, a healthy functioning confluent endothelial layer is often critical for the overall preservation of the implant or biomaterial. Here, we investigated the effects of microenvironmental factors, specifically substrate stiffness and adhesive cell ligands, on VEC stability and hemostatic function to inform the design of endothelialization strategies. The sensitivity of VEC hemostatic responses may be important in designing optimal endothelialization strategies for tissue engineered heart valves that can withstand physiological mechanical loads while protecting underlying cells and matrix from injury.

The adhesive cell ligands immobilized in each culture environment affected VEC adhesion and hemostatic regulation. VECs seeded on the modified RKR ligands had significantly higher levels of hemostatic protein expression than did VECs seeded on RGDS, both for thrombotic and anti-thrombotic proteins, exhibiting increased cellular activity. For example, VWF is a hyper-adhesive hemostatic protein secreted by stimulated or injured endothelial cells that mediates platelet adhesion to sites of endothelial injury when released in an activated ULVWF form [39,44]. Results from the gene expression PCR, showed that RKR-coated gels with seeded VECs produced higher levels of antigenic VWF and ADAMTS13. Although these cells produced more VWF according to the ELISA and immunohistological stain results, the fractions of VWF fragments between the RKR- and RGDS-coated hydrogels (3.4 kDa and 8 kDa) were not significantly different, suggesting that the increased level of VWF expression in VECs on RKR-coated hydrogels was accompanied by increased ADAMTS13 activity.

After introducing platelets to histamine-stimulated VECs on RKR scaffolds, the platelets adhered to the VEC-secreted/anchored ULVWF strings. In comparison, VECs grown on RGDS-coated scaffolds expressed and produced hemostatic proteins, but the mRNA expression levels for thrombotic proteins VWF and TF were significantly lower than for VECs grown on RKR and TCPS groups. Furthermore, the reduced level of VWF expression and release by the VECs on RGDS functionalized gels in comparison to RKR and TCPS groups is consistent with the significantly lower number of platelets adherent to the histamine stimulated VECs on RGDS-3.4 kDa constructs. These findings are relevant because healthy endothelium maintains a balanced production of various hemostatic molecules important in mediating blood clotting and platelet adhesion [1,2]. When endothelial damage occurs, which was simulated here by the addition of exogenous histamine, the balance of hemostatic protein expression shifts to a more pro-thrombotic phenotypes (i.e., increased VWF release and platelet adhesion) [1,2,39]. The pro-thrombotic shift during injury represent the expected physiologic VEC behavior as observed with the cells seeded on the RKR-immobilized hydrogels. Overall, the VECs cultured on RGDS-functionalized hydrogels required longer time to reach total confluence, showed difficulty in maintaining confluence in long term culture, had lower gene expression levels of both anti-thrombotic and thrombotic proteins during stable culture conditions (leaning toward anti-thrombotic), and demonstrated a reduced response to stimulation (significantly lower levels of released VWF and adhered platelets), suggesting an overall less healthy phenotype.

The pronounced effects of the adhesive ligands on VEC culture may be attributed to the specific adhesion receptors being engaged. RKR is a syndecan-1 and heparin binding ligand derived from the laminin-α1 globular G-domain [33,45]. In comparison, RGDS is a ubiquitous cell adhesive domain that is present in many ECM proteins, that promotes cell attachment via α_V_β_3_ or α_2_β_3_ integrin binding. Syndecan-1 is one of the major components of the endothelial glycocalyx [46], and maybe, therefore, highly abundant on VEC surfaces. Activation of synedcan-1 via ligand binding, such as RKR, primes the syndecan-1 ectodomain to couple with the β subunit of integrins, which promote a high affinity ligand binding state [47,48]. This conformational change may be sufficient for integrin activation [47], and may provide an explanation for the rapid adherence of VECs to RKR-coated gels while maintaining stable phenotype in comparison to the VECs less confluent adhesion to RGDS-coated gels. Though both syndecans and integrins have been shown to influence cell adhesion and function, our data indicates that VEC adhesion and microenvironmental responses could be more sensitive to syndecan-mediated binding and signaling, and warrants further investigation.

In addition to comparing VEC responses to specific adhesive ligands, we examined effects of substrate rigidity on VEC hemostatic functions. *In vivo*, the mechanical environment experienced by valves is sensed by and influences the resident cells that maintain the tissues. Moreover, previous work has shown that the mechanical support, modulated by the rigidity of ECM, strongly influences cellular behavior and plasticity [49]. Using hydrogels made from three different MWs provided a wide range of substrate stiffness to test VEC cultures and to compare with the commonly used stiffer materials such as TCPS (~50–90 GPa). RKR provided an advantage in promoting VEC adhesion to all substrate stiffness, including the softest 20 kDa (7 kPa) hydrogels. VECs on all RKR hydrogel compositions were able to form a confluent monolayer within a few days of culture, similar to VECs seeded on TCPS. However, VECs on the softer 8 kDa (35 kPa) and 20 kDa (7 kPa) RKR hydrogels had significantly more gene expression (> 50x) for all hemostatic-related proteins relative to VECs on TCPS, which suggests that softer substrates in combination with appropriate cell adhesive ligands promotes a robust, balanced VEC hemostatic capacity. In contrast, the RGDS ligand supported VEC attachment on hydrogels that were soft (compressive modulus 35 kPa), but overall had reduced hemostatic response levels than all RKR-coated gel conditions, which suggests that VEC adhesion and activation of the α_v_β_3_ and other integrins alone may be insufficient to support healthy VEC hemostatic regulation. Thus, it appears necessary to develop modulating microenvironments with appropriate adhesive ligands and substrate rigidity when attempting to develop a functional anti-thrombotic endothelium on cardiovascular implants.

In this study, we developed a simple, yet highly adaptable biomaterial platform to promote successfully stable VEC monolayer formation *in vitro* and characterize microenvironmental effects on VEC hemostatic regulation. PEGDA hydrogels are biocompatible, have easily tunable mechanical properties, and do not allow non-specific protein and cell adsorption. In addition to providing a modifiable substrate for cell culture experiments, PEGDA-based scaffolds are attractive biomaterials for tissue engineering and surface modification applications. Exploiting the available acrylate groups also allows simple covalent immobilization of thiolated adhesive ligands throughout the entire hydrogel surface. These methods and surface modification techniques can, therefore, be integrated into more complex 3D tissue engineered heart valve or cardiovascular endothelialization designs. Furthermore, adjustment of this culture platform could allow for *in vitro* modeling of valve disease states, especially in regards to mimicking pathological changes in environmental rigidity (e.g., fibrotic valves are stiffer and myxomatous valves become more compressible) [15,50].

In summary, after exposing VECs to various substrate stiffness and adhesive ligand combinations, we show that VEC stability and hemostatic protein expression is modulated by environmental factors such as specific ECM-substrate rigidity combinations. To our knowledge, this is the first report of the use of the RKR peptide sequence in combination with a PEGylated platform to promote stable endothelium formation. The efficiency of syndecan-1 binding to RKR ligands promoted VEC adhesion and proliferation on all substrates rigidities. The balanced and reactive nature of VECs seeded on the RKR immobilized hydrogels suggests preservation of a physiological and functional phenotype *in vitro*. Combinations of integrin (RGDS) and syndecan (RKR) mediated cell adhesion ligands on this platform may activate synergistic aspects of both pathways. Consideration of additional adhesive ligand domains from regional ECM proteins from non-integrin pathways warrants further investigation. Furthermore, the influence of mechanical stimulation may affect VEC hemostatic functions [51,52]. Investigation of these cell-adhesive ligands in combination with active mechanical stimulation such as shear stress and stretch also warrants characterization. Utilization of basement membrane-derived adhesion peptides along with softer substrate stiffnesses demonstrated the importance of environmental factors influencing phenotype of VECs, and may be important in valve implant endothelialization strategies.

## Supporting Information

S1 Fig Saturation curve of immobilized thiol-PEG-FITC on PEGDA hydrogels. Fluorescent intensity of immobilized of thiol-PEG-FITC with increasing initial concentrations added. Saturation occurs at initial thiol-PEG-FITC concentrations of 3 mM on 20 kDa PEGDA hydrogels and 5 mM on 3.4 kDa hydrogels.
(TIFF)

S1 Table Summary of hemostatic protein DNA primer sequences used for qRT-PCR. (DOCX)
