# Supplementary figures and images for: Laminin Peptide-Immobilized Hydrogels Modulate Valve Endothelial Cell Hemostatic Regulation
Source: PLoS One. 2015 Jun 19;10(6):e0130749. doi: 10.1371/journal.pone.0130749 (PMC4474637; doi:10.1371/journal.pone.0130749)

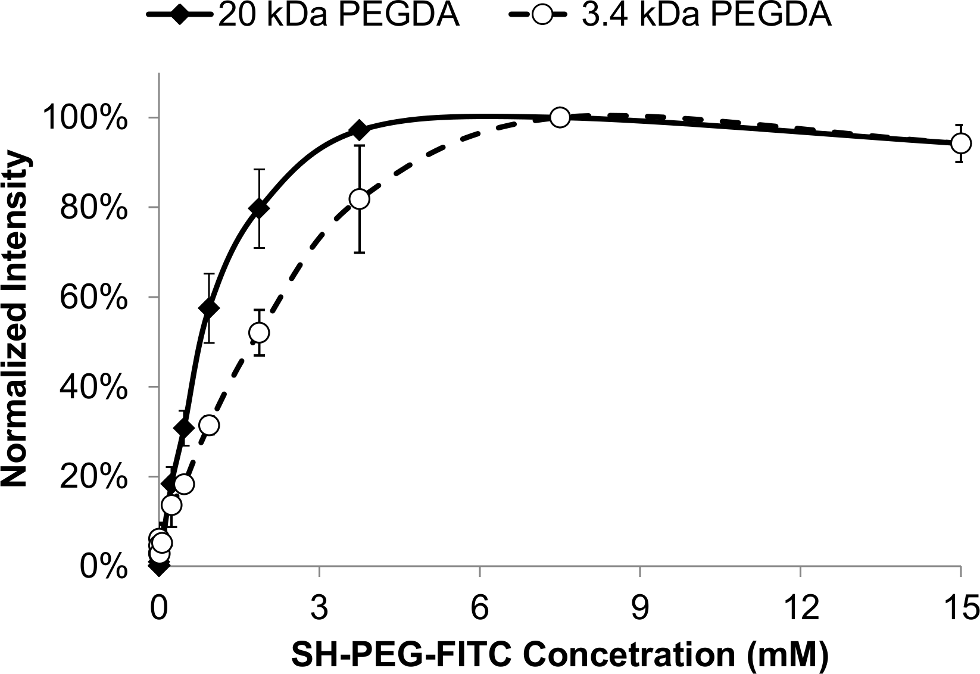

Supplement: S1 Fig — Fluorescent intensity of immobilized of thiol-PEG-FITC with increasing initial concentrations added. Saturation occurs at initial thiol-PEG-FITC concentrations of 3 mM on 20 kDa PEGDA hydrogels and 5 mM on 3.4 kDa hydrogels. (TIFF) [file pone.0130749.s001.tiff]
