# Supplementary material for: Laminin Peptide-Immobilized Hydrogels Modulate Valve Endothelial Cell Hemostatic Regulation
Source: PLoS One. 2015 Jun 19;10(6):e0130749. doi: 10.1371/journal.pone.0130749 (PMC4474637; doi:10.1371/journal.pone.0130749)
Supplement: S1 Table — (DOCX) [file pone.0130749.s002.docx]

| **Gene** |  | **5’🡨 DNA Sequence 🡪 3’** | **Product Size** | **Accession Number** |
| --- | --- | --- | --- | --- |
| ADAMTS-13 | Forward | CTCTGTTTCCTGTGGGGATG | 97 | Q76LX8 |
|  | Reverse | CAAGTGCTGGCAGAAATCAG |  |  |
| TFPI | Forward | GACCTCTTACTGCTATTC | 138 | EU090729 |
|  | Reverse | ATCTCTTCATCATTGCTT |  |  |
| PAI-1 | Forward | GGGACAGAACTGGAGATA | 136 | Y11347 |
|  | Reverse | GTCTAAGAGGCAGATTCG |  |  |
| VWF | Forward | CGAACCCAAGAAGAGAAT | 108 | S78431 |
|  | Reverse | ATCACTTCCTCCACAAAC |  |  |
| TF | Forward | TACAAGAGTAGAATCCAT | 107 | AY50424 |
|  | Reverse | AAGTGTCTAATGCTAATG |  |  |
| tPA | Forward | TGCCATTGACAAACACAT | 102 | BK007995 |
|  | Reverse | AACACTCCTTCTCCATCA |  |  |
| eNOS | Forward | AGAGAATGGAGAGAGTTT | 104 | AY266137 |
|  | Reverse | TATTGAAGCGGATTTTGT |  |  |
| GAPDH | Forward | CATTGACCTCCACTACAT | 119 | AF017079 |
|  | Reverse | AGATGGTGATGGGATTTC |  |  |
